# Left Nonrecurrent Laryngeal Nerve: A Very Unusual Finding during Thyroid Surgery

**DOI:** 10.1155/2022/4632501

**Published:** 2022-03-16

**Authors:** Nicolas Galat Ahumada, Flavio Carneiro Hojaij, Caroline Cunico, Hugo Genki Kagawa Akahane, Cleverson Alex Leitão, Jorge Eduardo Fouto Matias

**Affiliations:** ^1^Department of Surgery, Complexo Hospital de Clínicas, Universidade Federal do Paraná, Curitiba, PR, Brazil; ^2^Faculdade de Medicina da Universidade de São Paulo, São Paulo, SP, Brazil; ^3^Complexo Hospital de Clínicas, Universidade Federal do Paraná, Curitiba, PR, Brazil; ^4^Department of Radiology, Complexo Hospital de Clínicas, Universidade Federal do Paraná, Curitiba, PR, Brazil

## Abstract

**Background:**

Identifying the inferior laryngeal nerve is one of the main concerns in thyroid surgery. The typical recurrent position occurs due the relative position between the vagus nerve and the larynx during the last 3 branchial arches development. In rare cases, this nerve does not loop under the right subclavian artery or the aortic arch. This abnormality is present in 0.7% of patients and is associated with the presence of anatomical vascular anomalies. The left non-recurrent inferior laryngeal nerve is an even rarer abnormality, with only six cases described in the literature to date.

**Method:**

A 46- years old female patient referred to total thyroidectomy for symptomatic multinodular benign goiter.

**Results:**

A left non-recurrent inferior laryngeal nerve was found with difficulty and then a partial thyroidectomy was performed. CT scan showed dextroposition of the vessels of the base of the heart and an aberrant left subclavian artery.

**Conclusion:**

An association of a right-sided aortic arch and aberrant left subclavian artery, or the presence of situs inversus, although rare anatomical variations, are associated to a non-recurrent inferior left laryngeal nerve. Proper identifying these abnormalities may help to properly identify and salvage this structure.

## 1. Introduction

The inferior laryngeal nerve is a branch of the vagus nerve, which, in 99% of the cases, ascends towards the tracheoesophageal groove in the upper part of the chest, the other 1% being nonrecurrent. Its iatrogenic dysfunction is among the most feared complications of cervical explorations, as it is associated with temporary or permanent dysfunction of the vocal folds. Its injury can lead to changes in phonation and dyspnea. When the lesion is bilateral, the situation is even more drastic, usually requiring a tracheostomy as initial treatment. Thus, before cutting the branches of the inferior thyroid artery, it is essential to properly identify the inferior laryngeal nerve, its path, and anatomical variations, in order to avoid injuries to this structure. Reoperations or locally advanced malignant diseases impose even greater risks [1].

The first case of a nonrecurrent inferior laryngeal nerve was reported in 1823 by Stedman, who described the variation of a nonrecurrent nerve on the right side and identified an aberrant ipsilateral subclavian artery, running posterior to the esophagus, the so-called *arteria lusoria*. [2]. In 1935, an alleged abnormality of the left inferior laryngeal nerve was described, which, in fact, looped under a fibrotic structure described as the remnant of the ductus arteriosus, and was associated with aortic dextroposition and a retroesophageal left subclavian artery [3]. In 1985 Henry et al., two patients were first reported with the left nerve abnormality, *situs inversus*, and a retroesophageal left subclavian artery [4].

We report the case of a patient with cardiac dextroposition and retroesophageal left subclavian artery and with intraoperative finding of a left nonrecurrent inferior laryngeal nerve. We also review the cases of left nonrecurrent inferior laryngeal nerves published in the literature to date.

## 2. Case Presentation

A 46-year-old female patient was admitted for total thyroidectomy for nontoxic multinodular substernal goiter with compressive symptoms. The patient had a history of progressive growth of the anterior cervical region for about seven years. There was discomfort when swallowing regular oral diet, with progressive worsening in recent years and mild dyspnea. There were no voice impairment and no other associated specific symptoms. Laboratory tests revealed euthyroidism. The patient presented altered cognitive development since childhood; no other comorbidities and no alterations were found in the physical examination.

**Figure 1:**
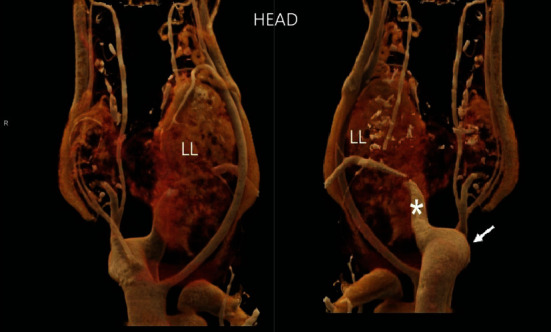
CT scan three-dimensional reconstruction. Frontal and posterior views of the cervical region, showing the volumetric increase in the thyroid, pronounced on the left lobe (LL), and evidencing the presence of the aortic arch on the right side (arrow), with left subclavian artery with retroesophageal path (asterisk).

A contrast enhanced neck CT scan identified a heterogeneous multinodular goiter, asymmetrically distributed by the thyroid gland, with hypodense center and hyperdense edges, with one nodule on the right of 44 cm^3^, another on the isthmus, with an approximate volume of 19.1 cm^3^, and a larger one in the left lobe, with an approximate volume of 124 cm^3^. The left-positioned mass compressed the larynx, decreased the right common carotid artery caliber by lateral displacement of the structures of the carotid space, deflected the trachea to the right narrowing its lumen, and took the upper part of the mediastinum between the trachea and the left brachiocephalic vein. There was no sign of esophageal displacement or compression. Three-dimensional CT reconstruction showed the presence of the aortic arch located on the right side, with left subclavian artery with retroesophageal path (Figure 1). No lymph node enlargement was found, as well as no alteration in the parotid and submandibular glands, and other cervical vessels.

Fine-needle aspiration (FNA) biopsy resulted in benign goiter with cystic degeneration, Bethesda II. However, due to the symptomatology and progressive worsening, a total thyroidectomy was scheduled.

**Figure 2:**
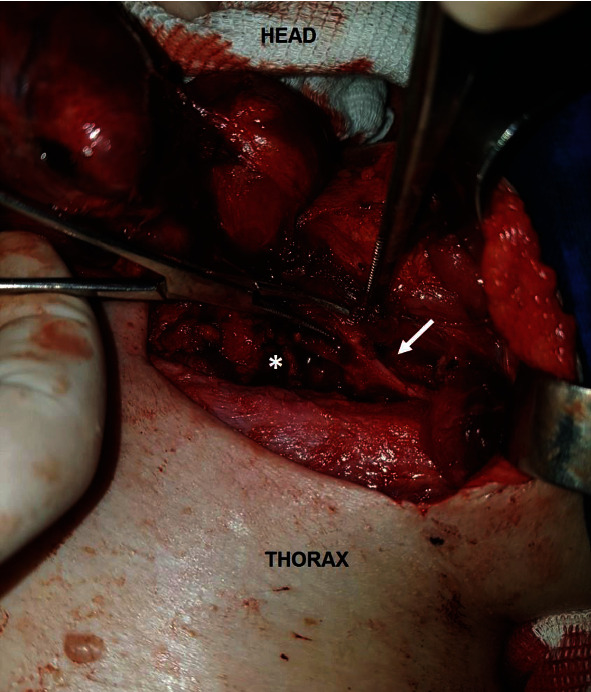
Surgical cervical exploration on the left nerve. Laryngeal nerve (arrow) out tracheoesophageal groove (asterisk), its usual anatomical position.

**Figure 3:**
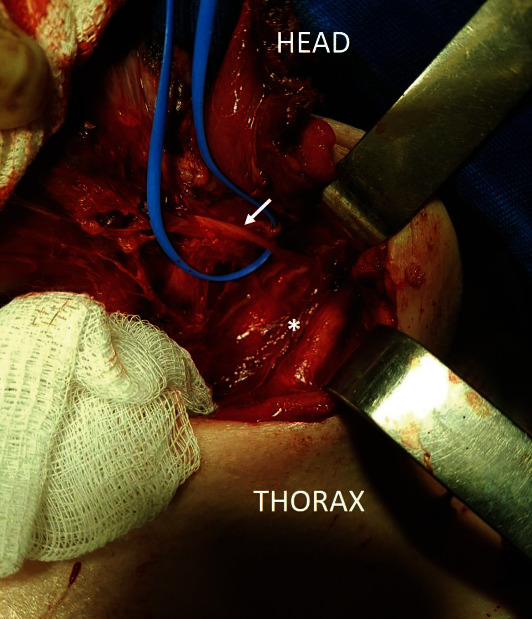
Dissection of the path of the left laryngeal nerve (arrow) until its emergence in the vagus nerve, in the dissected carotid sheath (asterisk). The left laryngeal nerve may be seen to follow a perpendicular path towards the thyroid (already resected in the photo above), as opposed to looping under the aortic arch.

During the procedure, the left nerve could not be identified in its usual position in the tracheoesophageal groove during cervical exploration (Figure 2). Recurrent laryngeal nerve monitoring was not available. Therefore, it was necessary to identify the nerve as it entered the larynx. Retrograde dissection toward the vagus nerve was performed. The left inferior laryngeal nerve was traced stemming from the medial segment of the vagus nerve, following a perpendicular path towards the thyroid, not recurring under the aortic arch (Figure 3). As it was possible to identify its entire path, it suffered no iatrogenic lesion. For safety reasons, the surgical team chose to limit the resection to the left lobe and isthmus, leaving the right nerve and lobe preserved, and after recovery assessment, a second surgery would be performed. The patient had good postoperative recovery and was discharged in good condition on the third day, accepting oral diet and without changes in phonation. The previously reported dyspnea and dysphagia were absent. The surgical piece weighed 146 g; in the anatomopathological analysis, it was shown to be compatible with colloid goiter and no malignancy was detected.

After surgery, outpatient follow-up was performed with annual laboratory tests and cervical ultrasound, and as the patient remained asymptomatic and euthyroid, the surgical team and patient chose to not perform a second operation. The annual ultrasonography showed a topical right lobe, with cystic areas and calcifications of permeation, with the largest cyst measuring 2.5 cm and a glandular volume of approximately 50 cm^3^, with insignificant volumetric variations between examinations.

## 3. Discussion

In a systematic review, Ling et al., in a total of 38,568 inferior laryngeal nerves, found 221 reports of nonrecurrent inferior laryngeal nerves (0.57%) [5]. A meta-analysis published in 2017 surveyed papers describing a total 33,571 laryngeal nerves on the right and 20,006 on the left. There was a prevalence of 0.7% of nonrecurrent laryngeal nerves on the right, 86.7% of which were associated with the aberrant right subclavian artery. The left nonrecurrent abnormality was found in only one study that described two patients, representing a prevalence close to 0%, with statistical difference [6]. In the present literature review, six left nonrecurrent inferior laryngeal nerves were identified (Table 1).

The first anatomical description of the inferior laryngeal nerve was performed by Galen of Pergamon in the second century as a branch off the cranial nerves. Lahey and Hoover described the importance of its mandatory identification and dissection in the surgical technique of thyroidectomy [7]. After the first description by Berlin in 1935 [3], it took fifty years for another left nonrecurrent inferior laryngeal nerve to be described, this time in two patients with complete situs inversus and left retroesophageal subclavian artery [4]. Fellmer et al. question the authenticity of these two reports as “true” left nonrecurrent inferior laryngeal nerves, since they are associated with situs inversus viscerum, thus constituting a mirror image of the right recurrent laryngeal nerve. In their paper, a left nonrecurrent inferior laryngeal nerve was described in a patient with right-sided aorta*, truncus arteriosus communis*, and an aberrant left innominate artery [8]. In addition to these, two recent papers reported one case each in patients with right-sided aortic arch and aberrant left subclavian artery [9, 10].

The crucial point noted in all nonrecurrent laryngeal nerves, whether on the left or right side, is the presence of the so-called *arteria lusoria*, that is, an aberrant subclavian artery, the course of which is posterior to the esophagus, occasionally leading to dysphagia lusoria. Therefore, knowledge of the possible anatomical variations should be considered, especially in the preoperative stage, as dysphagic symptoms may not be caused by thyroid enlargement, but by the presence of this anatomical abnormality, with no likely symptomatic improvement during the postoperative period [8].

Left nonrecurrent inferior laryngeal nerves are very rare, as this abnormality requires the presence of a combination of rare vascular malformations. Three factors are required for this nerve to be positioned on the left: the presence of an aortic arch on the right, an aberrant right subclavian artery, and the absence of the ductus arteriosus or its remnants [11].

Embryologically, the inferior laryngeal nerve recurrence is closely associated with the development of the fourth, fifth, and sixth branchial arches on both sides. As the fifth and sixth arches on the right side are reabsorbed during embryogenesis, the right inferior laryngeal nerve can only loop under a fourth arch structure (right subclavian artery). On the left side, the nerve loops around both the fourth arch (the adult aortic arch) and the sixth arch (the ductus arteriosus) as the fifth arch is normally regressed. The development of a left nonrecurrent inferior laryngeal nerve requires additional regression of both the fourth and sixth primitive aortic arches. As such, a right-sided aorta is present because the right fourth arch is the only one responsible for its origin [10].

In 2018, Hua et al., after careful assessment of the patient vascular anatomy, successfully predicted the presence of a nonrecurrent left laryngeal nerve prior to a total thyroidectomy for a patient with Graves disease [10]. However, Masuoka et al. in 2016, in a report of four patients previously diagnosed with the corresponding vascular anomalies, only found one nonrecurrent inferior left laryngeal nerve [9]. Both patients reported in these recent papers had a chromosome 22q11 defect [9, 10]. Chromosome 22q11 deletion is associated with isolated anomalies of laterality or branching of the aortic arch in 24% in a series of cases published by McElhinney et al. [12].

The main complications of thyroidectomies involve the injury of adjacent structures, in particular the lesion of the inferior laryngeal nerves and the parathyroid glands. The rate of nerve injury varies according to the thyroid disease (0.2%–25.0%), the presence of previous procedures (primary surgery (0.6%)–reoperation (3.6%)), the extent of thyroid resection (partial (0.7%)–total (1.3%)), and the surgeon's experience (0.6%–1.4%). Despite the technological advent of intraoperative neuromonitoring, visual identification of the inferior laryngeal nerve remains the gold standard in the prevention of lesions to the inferior laryngeal nerve, preventing hoarseness due to paralysis of the vocal folds in the postoperative period in unilateral lesions, and respiratory failure in bilateral lesions [13]. It was observed that cases of right nonrecurrent inferior laryngeal nerves present a much more oblique, even more transverse course than anatomically expected [1].

Despite the rare occurrence of nonrecurrent inferior laryngeal nerve, knowledge about the profile of patients who are likely to present the anatomical abnormalities leading to its development, its prevalence, and variations in anatomical patterns may enable surgeons to infer an anatomical characterization that leads to greater surgical safety.

## Figures and Tables

**Table 1:** Cases reported in the literature describing nonrecurrent inferior laryngeal nerve on the left.

Author	Year	Associated abnormalities	Cases described
Berlin	1935	Right-sided aortic arch Retroesophageal left subclavian artery Remaining ductus arteriosus	1
Henry	1985	*Situs inversus viscerum* Retroesophageal left subclavian artery	2
Fellmer	2008	Right-sided aortic arch *Truncus arteriosus communis* Aberrant left innominate artery	1
Masuoka	2016	Right-sided aortic arch Aberrant left subclavian artery	1
Hua	2018	Right-sided aortic arch Aberrant left subclavian artery	1
Ahumada	2021	Right-sided aortic arch Aberrant left subclavian artery	1
Total of patients described in the literature			7

## Data Availability

Previously reported cases were used to support this study and are available and cited at relevant places within the text as references.
